# Identification of prognostic nutritional index as a reliable prognostic indicator for advanced lung cancer patients receiving immune checkpoint inhibitors

**DOI:** 10.3389/fnut.2023.1213255

**Published:** 2023-07-28

**Authors:** Xuebing Yan, Jiaxin Wang, Jingxian Mao, Ying Wang, Xiangjun Wang, Mengxue Yang, Hong Qiao

**Affiliations:** ^1^Department of Oncology, The Affiliated Hospital of Yangzhou University, Yangzhou University, Yangzhou, China; ^2^Department of Oncology, Hefei Cancer Hospital, Chinese Academy of Sciences, Hefei, China; ^3^Department of Oncology, Baoying Traditional Chinese Medicine Hospital, Yangzhou, China

**Keywords:** lung cancer, prognostic nutritional index, immune checkpoint inhibitors, prognosis, nomogram

## Abstract

**Background:**

Prognostic nutritional index (PNI) has been identified as a reliable prognostic factor for cancer adjuvant therapy. However, its prognostic value in lung cancer patients receiving immune checkpoint inhibitors (ICIs) remains inconclusive.

**Method:**

A systematic literature review and meta-analysis was performed based on online databases before March 1th 2023. The correlation of PNI with overall survival (OS) or progression-free survival (PFS) was determined using the hazard ratios (HRs) coupled with 95% confidence intervals (CIs). Then, a retrospective cohort enrolling 123 ICI-treated lung cancer patients from two hospitals was utilized for validation and further investigation.

**Results:**

A total of 14 studies enrolling 1,260 lung cancer patients were included in the meta-analysis. The high PNI level was significantly correlated with better OS (HR = 2.56, 95% CI = 1.86–3.54) and PFS (HR = 1.91, 95% CI = 1.53–2.40) of the lung cancer patients. The subgroup analysis confirmed the results except for the PFS in patients receiving anti-PD-1 therapy (HR = 1.51, 95% CI = 0.86–2.65). In the retrospective study, the high PNI level was identified as a favorable factor for OS and PFS not only in the whole cohort but also in the subgroups stratified by non-small cell lung cancer and small cell lung cancer. The high PNI was also correlated with better anti-cancer therapy response and performed better than body mass index and serum albumin level in OS prediction. Finally, we established a novel prognostic nomogram based on PNI and other clinical parameters. The nomogram was found to perform well in predicting the 1-year OS of ICI-treated lung cancer patients.

**Conclusion:**

Both the meta-analysis and retrospective work demonstrate the PNI is a reliable prognostic factor for advanced lung cancer patients receiving ICI-based therapies. Our study further highlights the crucial role of nutrition assessment and intervention in cancer immunotherapy.

**Systematic review registration:**

https://www.crd.york.ac.uk/prospero/, identifier: CRD42023424146.

## Introduction

Lung cancer is the second most frequently diagnosed human malignancy worldwide, ranking the first for cancer mortality among cancers (1). Despite advances in tobacco control and pre-cancer screening, the 5-year relative survival rate for lung cancer is only ~21–23% (2). Targeted therapies based on sequencing have dramatically improved the prognosis of lung cancer patients, however, drug resistance commonly occurs (3). Moreover, the therapeutical strategies for advanced lung cancer without gene mutation were extremely limited. The immune checkpoints inhibitors (ICIs) have shown promise in durable tumor control through taking advantage of host immunity (4). However, in addition to genetic background, various clinical factors were found to affect the actual efficacy of ICIs. For instance, our team previously had proved the correlation between concomitant medications (antibiotics, corticosteroids, proton pump inhibitor and β-blockers) and ICI efficacy (5–8). Therefore, further investigations into the impact of clinical factors on ICI efficacy is urgent and will contribute to more precise therapy decision.

Among lung cancer patients, ~70% are found to suffer from malnutrition or muscle loss, suggesting the crucial role of nutrition assessment during anti-cancer therapy (9). Traditional nutrition related markers such as body mass index (BMI) and albumin (ALB) level have been utilized to predict lung cancer risk or patient survival (10, 11). The prognostic nutritional index (PNI), calculated by serum albumin level and lymphocyte count, has recently been identified as a novel easy-to-perform and standardized tool for prognosis prediction in various cancers (12). In lung cancer, pretreatment PNI has been found to associate with the clinical efficacy of targeted therapy, chemotherapy and radiotherapy (13–15). With regard to ICI-based immunotherapy, in a small cohort of non-small cell lung cancer (NSCLC) patients treated with atezolizumab (*n* = 24), high PNI level was correlated with prolonged time to treatment failure and overall survival (OS) (16). The pre-treatment PNI was identified as an independent prognostic factor for OS instead of progression-free survival (PFS) in advanced non-small cell lung cancer (NSCLC) patients receiving anti-PD-1 therapy (17). A recent comprehensive study has investigated the prognostic value of PNI and inflammatory indexes in immunotherapy-treated lung cancer patients (18). The univariate analysis in this study demonstrated high PNI level was correlated with prolonged PFS instead of OS, and the multivariate analysis failed to identify it as an independent prognostic factor. Therefore, the prognostic impact of PNI in ICI-treated lung cancer patients remains inconclusive and more clinical validations are essential.

In this study, firstly, a systematic literature review and meta-analysis was performed to objectively assess the prognostic value of PNI in lung cancer patients receiving ICI therapy. Then, a retrospective cohort enrolling 123 lung cancer patients from two hospitals was utilized to confirm the prognostic value of PNI and establish a novel PNI-based prognostic nomogram. This study will not only contribute to the establishment of a non-invasive and low-cost predictive approach for ICI therapy, but also further highlight the crucial role of nutrition assessment and intervention in patient management.

## Materials and methods

### Search strategy for literature search

The systematic review and meta-analysis was carried out according to the guidelines of Preferred Reporting Items for Systematic Reviews and meta-Analyses (PRISMA). The literatures related with the prognostic role of PNI in ICI-treated lung cancer patients were searched in PubMed, Embase, Cochrane Library, Web of Science, and Clinicaltrials.gov. The deadline for literature publication date was March 1st 2023. The following key words were used for literature search: prognostic nutritional index, PNI, lung, cancer, tumor, neoplasm, programmed death receptor 1 inhibitor, PD-1 inhibitor, programmed death-ligand 1 inhibitor, PD-L1 inhibitor, immunotherapy, cytotoxic T lymphocyte antigen-4 inhibitor, and CTLA-4 inhibitor. For avoiding missing the eligible studies, the relevant literatures or meeting abstracts published in the supplementary issues were carefully reviewed.

The inclusion criteria for literature search were as follows: (1) lung cancer patients; (2) ICI therapy with or without other standard therapies such as chemotherapy, radiotherapy and targeted therapy; (3) case-control or cohort studies containing the high-PNI and low-PNI group; (4) available hazard ratios (HRs) with 95% confidence intervals (CIs) for OS and/or PFS. The exclusion criteria were as follows: (1) duplicate reports; (2) irrelevant topics; (3) meta-analysis or reviews; (4) non-clinical studies; (5) lack of key patient information.

**Figure 1:**
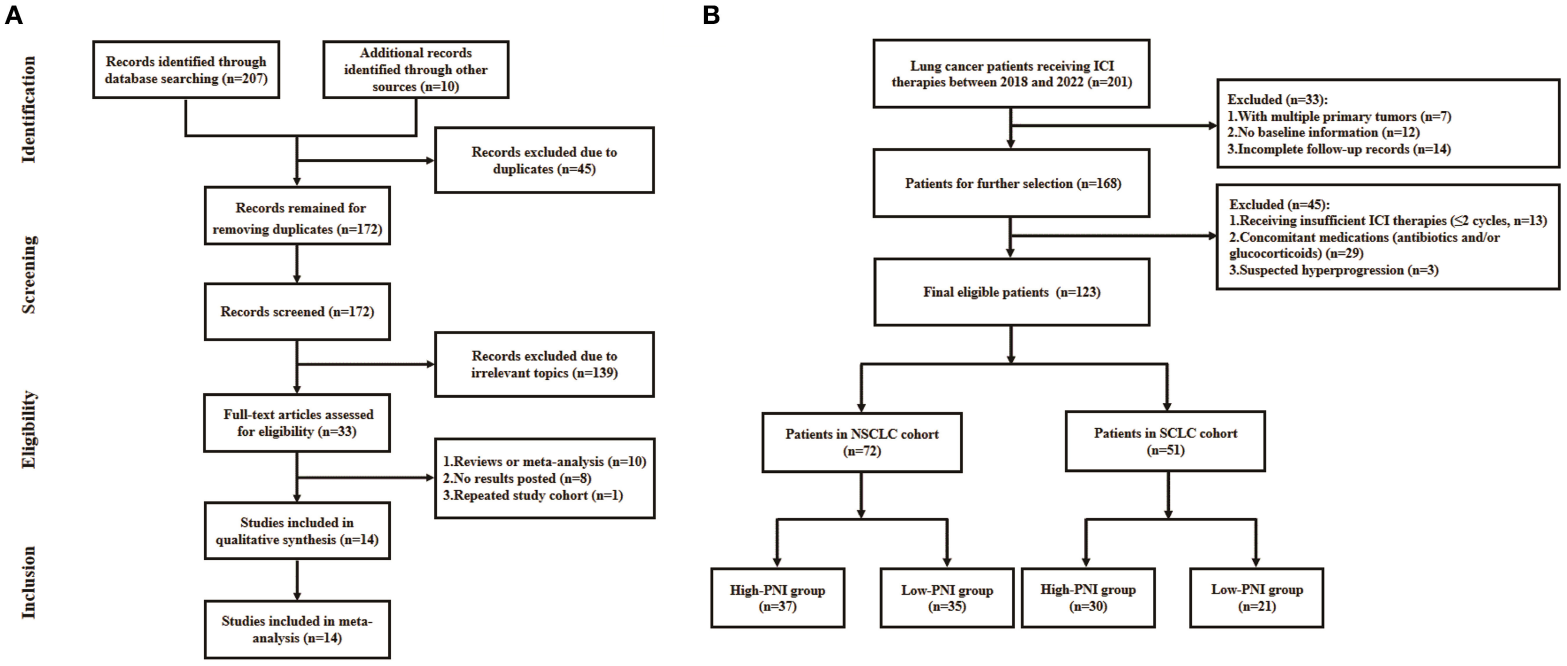
Flowchart of the literature search for meta-analysis **(A)** and patient recruitment in the retrospective study **(B)**.

The flowchart of literature search was shown in Figure 1A. A total of 217 literatures were initially selected from online databases and other sources. Then, the literatures were excluded due to duplications, irrelevant topics, meta-analysis or review, no posted result and repeated study cohorts. Finally, a total of 14 studies were included in the meta-analysis.

### Data extraction and quality assessment

As shown in Table 1, the following information was extracted from the included studies: author, country, sample size, median age, cut-off value of PNI, anti-cancer treatment and survival data. In case that the HRs of the univariate and multivariate analysis were available, the latter were selected to minimize the impact of confounding factors. In case that the HRs were not provided directly in the literatures, they were estimated using the method described in a previous study (19). The literature quality was assessed using the Newcastle-Ottawa Scale. The literature scored no <6 points was considered to be high-quality.

**Table 1:** Baseline characteristics of the included studies in the meta-analysis.

**References**	**Country**	**Number**	**Age (year)**	**Cut-off**	**Tumor type**	**Treatment**	**OS**	**PFS**	**NOS**
							**HR (95% CI)**	**HR (95% CI)**	
Shoji et al. (23)	Japan	102	69	45.5	NSCLC	PD-(L)1 monotherapy/ combination therapy	1.606 (0.952–2.745)	1.704 (1.039–2.828)	8
Cipriano et al. (22)	Portugal	34	67	50	NSCLC	NA	6.67 (1.45–33.3)	NA	7
Matsubara et al. (16)	Japan	24	64.5	48	NSCLC	PD-L1	7.28 (0.92–57.4)	NA	7
Peng et al. (24)	China	102	62	45	NSCLC	PD-1	2.79 (1.57–4.95)	1.92 (1.14–3.25)	8
Baldessari et al. (25)	Italy	44	70	45.1	NSCLC	PD-1	1.86 (0.76–4.56)	NA	7
Liu et al. (27)	China	123	59.9	46.05	NSCLC	PD-1 monotherapy/ combination therapy	7.222 (4.081–12.781)	2.698 (1.752–4.153)	8
Ogura et al. (28)	Japan	34	72	40	NSCLC	PD-(L)1 monotherapy/ combination therapy	1.80 (0.54–5.98)	1.59 (0.57–4.38)	7
Qi et al. (29)	Multicenter	53	NA	48	SCLC	PD-(L)1 combination therapy	0.88 (0.30–2.63)	NA	8
Shi et al. (30)	China	103	66	45	NSCLC	PD-(L)1/anti–CTLA4 monotherapy/ combination therapy	3.40 (1.42–8.13)	2.47 (1.12–5.43)	8
Cipriano et al. (26)	Portugal	52	NA	50	NSCLC	Pembrolizumab, nivolumab, and atezolizumab	2.86 (1.38–5.95)	3.01 (1.47–6.20)	7
Tanaka et al. (33)	Japan	237	69	40.35	NSCLC	PD-(L)1 monotherapy/ combination therapy	2.38 (1.23–4.62)	2.01 (1.28–3.15)	8
Zaitsu et al. (18)	Japan	95	70.9	43	NSCLC	Nivolumab, pembrolizumab, and atezolizumab	0.98 (0.31–3.13)	0.93 (0.38–2.24)	8
Shijubou et al. (31)	Japan	38	75	40	NSCLC	Pembrolizumab	NA	0.43 (0.13–1.52)	8
Stares et al. (32)	Scotland	219	69	45	NSCLC	Pembrolizumab	2.70 (1.75–4.17)	1.87 (1.30–2.69)	7

NSCLC, non-small-cell lung cancer; SCLC, small-cell lung carcinoma; ICI, immune checkpoint inhibitor; CTLA-4, cytotoxic T-Lymphocyte-associated Antigen 4; PD-1, programmed cell death protein-1; PD-L1, programmed cell Death-Ligand 1; OS, overall survival; PFS, progression-free survival; HR, Hazard ratio; CI, confidence interval; NA, not available.

### Patient recruitment of the retrospective clinical study

The lung cancer patients who received ICI therapies at the Department of Oncology, Hefei Cancer Hospital or Affiliated Hospital of Yangzhou University between January 2018 and October 2022 were recruited in the study. The inclusion criteria were as follows: (1) patients pathologically diagnosed as lung cancer; (2) patients receiving ICI-based anti-cancer treatments. The exclusion criteria were as follows: (1) multiple primary tumors; (2) lack of baseline information; (3) incomplete follow-up records; (4) patients receiving insufficient ICI therapy (no more than two cycles); (5) long-term administration of antibiotic and/or corticosteroids that were known to affect the ICI efficacy; (6) suspected hyperprogression. Finally, a total of 123 patients were included for analysis and the recruitment flowchart was shown in Figure 1B. The clinical characteristics of the cohort were shown in **Table 4**. This study was approved by the ethics committees of Affiliated Hospital of Yangzhou University (No. 2022-YKL11-05) and Hefei Cancer Hospital (No. PJ-KY2022-007). The written informed consents for using patient information in scientific researches were obtained from patients or their authorizers.

### Cut-off definition of PNI, BMI, and albumin level

The PNI was calculated as follows: PNI = albumin (g/L) + 5 × peripheral serum lymphocyte count (10^9^/L). The cut-off value of PNI in the retrospective clinical study was defined as the median value of PNIs in the meta-analysis (median value = 45.05, Table 1). The BMI was calculated as follows: BMI = weight (Kg)/height (m)^2^. The cut-off values of BMI and albumin (ALB) level were defined as 25 kg/m^2^ and 35 g/L respectively according to previous studies (20, 21).

### Oncological assessment of the retrospective clinical study

Oncological assessment was performed based on radiological examination and tumor marker detection every two or three cycle. The Response Evaluation Criteria in Solid Tumors (RECIST) 1.1 criteria was utilized to assess the tumor responses to ICI-based therapies, which was classified as complete response (CR), partial response (PR), progressive disease (PD) and stable disease (SD). The OS and PFS were used for outcome assessment, where OS was defined as the time period from the first ICI therapy to the death from any cause and PFS was defined as the time period from the first ICI therapy to disease progression.

### Statistical analysis

The meta-analysis was performed using the Stata SE 14.0 software. The Cochran *Q*-test and Higgins inconsistency index (*I*^2^) were utilized to determine the study heterogeneity. The sensitivity analysis was used to assess the result stability. The Begg's and Egger's test was used to assess the publication bias. In the retrospective clinical study, the statistical analysis was carried out using SPSS 21.0 statistical software and the figures were created using GraphPad Prism 8.0 software. The correlations of PNI with clinical characteristics were determined by the chi-squared test. The survival curves were created using the Kaplan-Meier model and the difference between the survival curves were analyzed using the log rank test. The independent prognostic factors were identified using the univariate and multivariate analysis, and the results were visualized using the R 4.2.1 software. A *p*-value < 0.05 was considered to be statistically significant.

## Results

### General characteristics and quality assessment of the included studies in the meta-analysis

A total of 14 studies were included in the meta-analysis and the details were shown in Table 1 (16, 18, 22–33). Majority of the patients were diagnosed as NSCLC with their median age ranging from 59.9 to 75 years old. The cut-off values of the PNIs ranged from 40 to 50. All the included studies were retrospective work, among which OS and PFS data were unavailable in one and four studies, respectively. According to the Newcastle-Ottawa Scale, all the included literatures were scored more than six points, suggesting the high quality of our data sources.

### Prognostic impact of PNI in the lung cancer patients from the included studies

**Figure 2:**
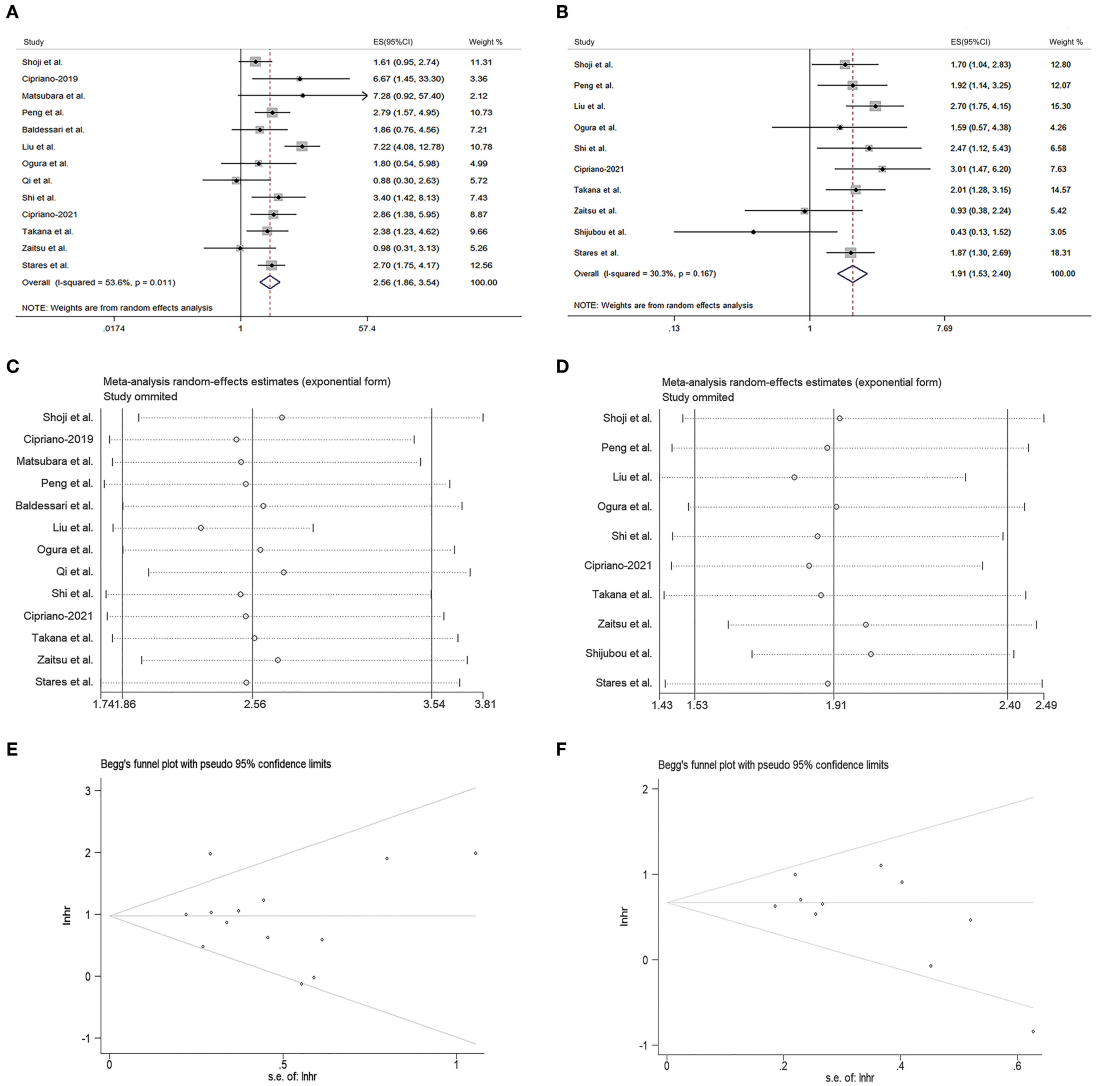
Prognostic significance of prognostic nutritional index (PNI) in the meta-analysis. **(A, B)** Forest plots of the hazard ratios (HRs) for the correlations of PNI with overall survival (OS) **(A)** and progression-free survival (PFS) **(B)**. **(C, D)** Sensitivity analysis of the studies assessing OS **(C)** and PFS **(D)**. **(E, F)** Begg's funnel plots for evaluating publication bias of OS **(E)** and PFS **(F)**.

As shown in Figure 2A, the pooled meta-analysis demonstrated the PNI was positively associated with the OS of the ICI-treated lung cancer patients (HR = 2.56, 95% CI = 1.86–3.54, *I*^2^ = 53.6%, *p* = 0.011). The similar result was also observed in the PFS of the ICI-treated lung cancer patients (HR = 1.91, 95% CI = 1.53–2.40, *I*^2^ = 30.3%, *p* = 0.167, Figure 2B). The sensitivity analysis revealed both the results could not be significantly affected by any single study (Figures 2C, D). The Begg's test demonstrated no publication bias for the pooled HR values of OS (Figure 2E, *p* = 0.807) and PFS (Figure 2F, *p* = 0.128), as subsequently confirmed by the Egg's test (*p* = 0.754 for OS and *p* = 0.146 for PFS).

### Prognostic impact of PNI in the subgroups of the meta-analysis

As shown in Table 2, the positive correlation between PNI and OS was observed in all the subgroups of region (Asia: HR = 2.69, 95% CI = 1.68–4.32, No-Asia: HR = 2.70, 95% CI = 1.93–3.79), age (>65 year old: HR = 2.23, 95% CI = 1.71–2.89; ≤65 year old: HR = 4.75, 95% CI = 2.18–10.32), PNI cut-off value (≤45.05: HR = 2.52, 95% CI = 1.92–3.31; >45.05: HR = 2.81, 95% CI = 1.49–5.30) and therapy type (ICI drug: HR = 2.61, 95% CI = 1.98–3.46; anti-PD-1 drug: HR = 2.60, 95% CI = 1.88–3.59; combined therapy: HR = 2.61, 95% CI = 1.98–3.46). As shown in Table 3, the similar correlation between PNI and PFS was observed in all the subgroups of region (Asia: HR = 1.80, 95% CI = 1.35–2.40; No-Asia: HR = 2.14, 95% CI = 1.41–3.24), age (>65 year old: HR = 1.67, 95% CI = 1.27–2.21, ≤65 year old: HR = 2.35, 95% CI = 1.69–3.28), and PNI cut-off value (≤45.05: HR = 1.71, 95% CI = 1.29–2.26; >45.05: HR = 2.34, 95% CI = 1.67–3.27). In terms of therapy type, the PNI was positively correlated with PFS in the patients receiving ICI (HR = 1.60, 95% CI = 1.02–2.52) or combined therapy (HR = 2.14, 95% CI = 1.67–2.73), instead of those receiving anti-PD-1 drug alone (HR = 1.51, 95% CI = 0.86–2.65).

**Table 2:** Subgroup analysis of the correlation between PNI and overall survival.

**Subgroup**	**No. of studies**	**OS hazard ratios (95% CI)**	***p*-value**	**Heterogeneity**	
				*I* ^2^	***p*** **-value**
**Region**					
Asia	8	2.69 (1.68–4.32)	< 0.001	64.60%	0.006
No-Asia	4	2.70 (1.93–3.79)	< 0.001	0.00%	0.579
**Age group**					
≤ 65	3	4.75 (2.18–10.32)	< 0.001	63.60%	0.064
>65	8	2.23 (1.71–2.89)	< 0.001	3.500%	0.403
**Cut-off value**					
≤ 45.05	6	2.52 (1.92–3.31)	< 0.001	0.00%	0.614
>45.05	7	2.81 (1.49–5.30)	0.001	72.70%	0.001
**Therapy type**					
ICI	7	2.61 (1.98–3.46)	< 0.001	0.00%	0.450
PD−1	3	2.60 (1.88–3.59)	< 0.001	0.00%	0.732
Combination	6	2.61 (1.98–3.46)	0.005	75.10%	0.001

PD-1, programmed cell death protein-1; CI, confidence interval; ICI, immune checkpoint inhibitor.

**Table 3:** Subgroup analysis of the correlation between PNI and progression-free survival.

**Subgroup**	**No. of studies**	**PFS hazard ratios (95% CI)**	***p*-value**	**Heterogeneity**	
				*I* ^2^	***p*** **-value**
**Region**					
Asia	8	1.80 (1.35–2.40)	< 0.001	38.70%	0.121
No-Asia	2	2.14 (1.41–3.24)	< 0.001	25.30%	0.247
**Age group**					
≤ 65	2	2.35 (1.69–3.28)	< 0.001	0.00%	0.326
>65	7	1.67 (1.27–2.21)	< 0.001	27.00%	0.222
**Cut-off value**					
≤ 45.05	7	1.71 (1.29–2.26)	< 0.001	27.90%	0.215
>45.05	3	2.34 (1.67–3.27)	< 0.001	17.70%	0.297
**Therapy type**					
ICI	5	1.60 (1.02–2.52)	0.041	57.40%	0.052
PD-1	3	1.51(0.86–2.65)	0.153	61.90%	0.072
Combination	5	2.14 (1.67–2.73)	< 0.001	0.00%	0.657

PD-1, programmed cell death protein-1; CI, confidence interval; ICI, immune checkpoint inhibitor.

### Prognostic impact of PNI in an independent retrospective validation cohort

**Figure 3:**
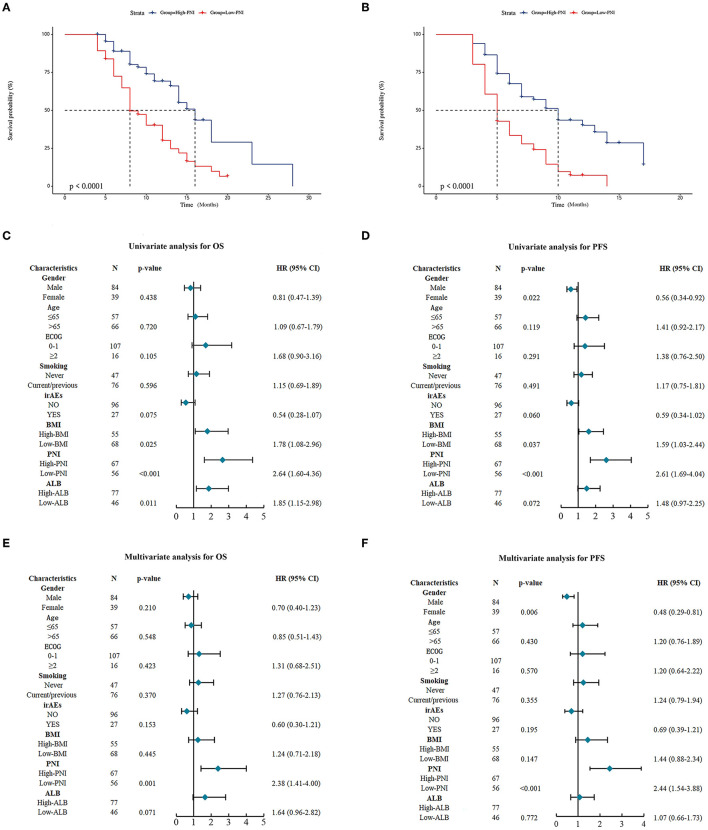
Prognostic significance of prognostic nutritional index (PNI) in the validation cohort. **(A, B)** Kaplan-Meier curves for the association of PNI with overall survival (OS) **(A)** and progression-free survival (PFS) **(B)** of the entire validation cohort. **(C, D)** Univariate analysis for identifying the factors significantly correlated with the OS **(C)** and PFS **(D)** of the entire validation cohort. **(E, F)** Multivariate analysis for identifying the significantly independent factors affecting OS **(E)** and PFS **(F)** of the entire validation cohort.

For validating the results of the meta-analysis, an independent retrospective cohort enrolling 123 lung cancer patients from two hospitals was utilized. As shown in Table 4, no significant correlation was found between the PNI level and gender, smoking status, ECOG PS scores, immuno-checkpoint inhibitors related adverse effects (iRAEs), BMI and ALB, except for age (*p* = 0.031). The survival analysis demonstrated the patients with high PNI level had a significant better OS and PFS than those with low PNI level (Figures 3A, B). The univariate analysis indicated the PNI level was significantly correlated with the OS and PFS of the lung cancer patients (Figures 3C, D). In addition, the multivariate analysis identified high PNI level as an independent favorable factor affecting both OS and PFS (Figures 3E, F).

**Table 4:** General characteristics of the validation cohort.

**Characteristics**	**PNI level**		**Total**	***p*-value**
	**High**	**Low**		
Age				**0.031**
≤ 65	37	20	57	
>65	30	36	66	
Gender				0.924
Male	46	38	84	
Female	21	18	39	
Smoking status				0.823
Never	25	22	47	
Current/previous	42	34	76	
ECOG PS				0.878
0-1	58	49	107	
≥2	9	7	16	
irAEs				0.060
No	48	48	96	
Yes	19	8	27	
Body mass index				0.457
≥25	32	23	55	
< 25	35	33	68	
ALB				0.058
≥35	47	30	77	
< 35	20	26	46	

ECOG PS, eastern cooperative oncology group performance status; irAEs, immune-related adverse events; ALB, albumin.

The bold values indicate *p* value < 0.05.

### Prognostic impact of PNI in the NSCLC or SCLC patients from the validation cohort

**Figure 4:**
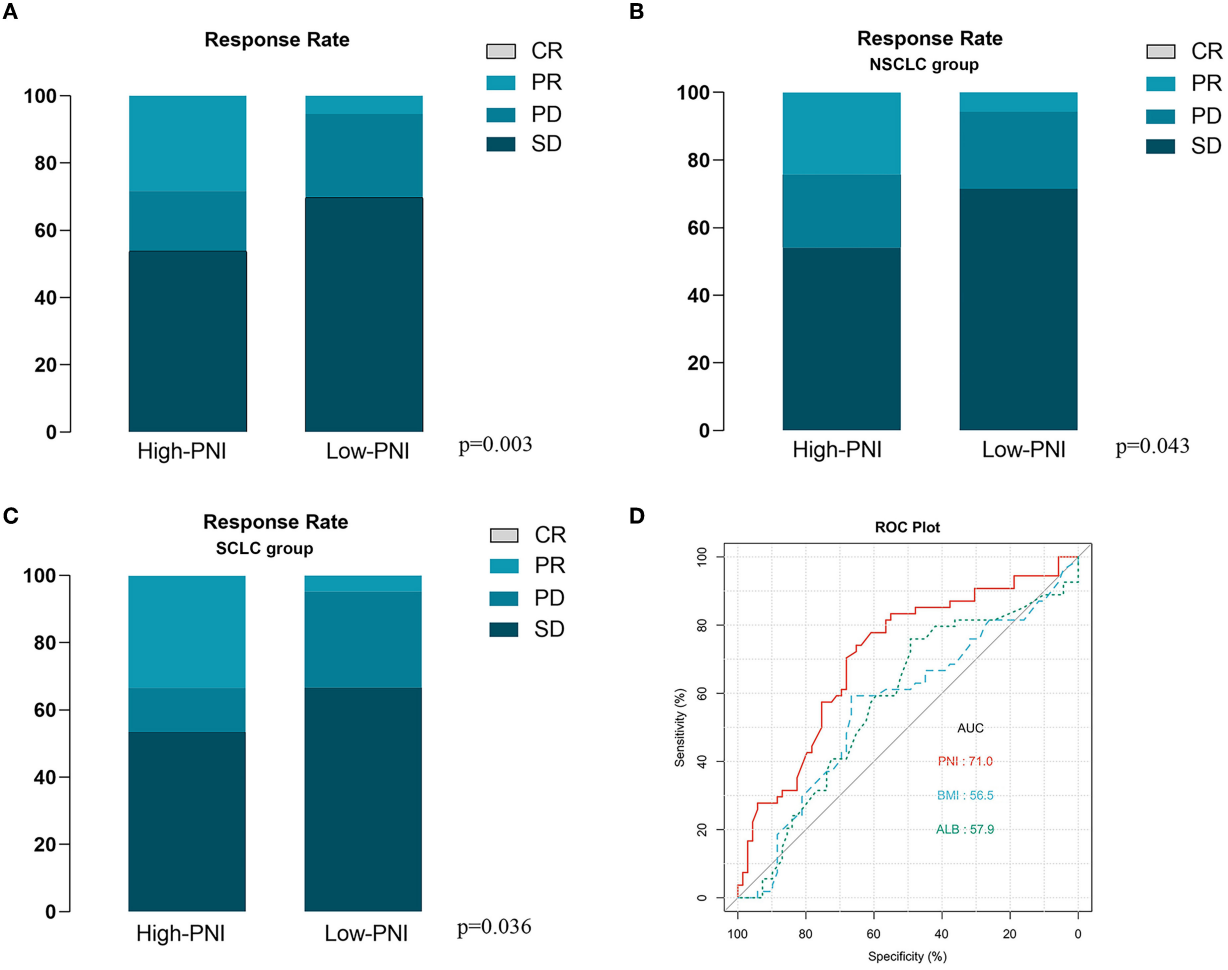
Correlation of prognostic nutritional index (PNI) with immune checkpoint inhibitor (ICI) based therapy response. **(A)** The high PNI level is significantly correlated with better therapy response in the entire validation cohort. **(B, C)** The high PNI level is significantly correlated with better therapy response in patients with NSCLC **(B)** and SCLC **(C)**. **(D)** The receiver operator characteristic (ROC) curve model was used to compare the predictive performance of PNI, Body Mass Index (BMI) and albumin level in the overall survival (OS) of the entire validation cohort.

**Figure 5:**
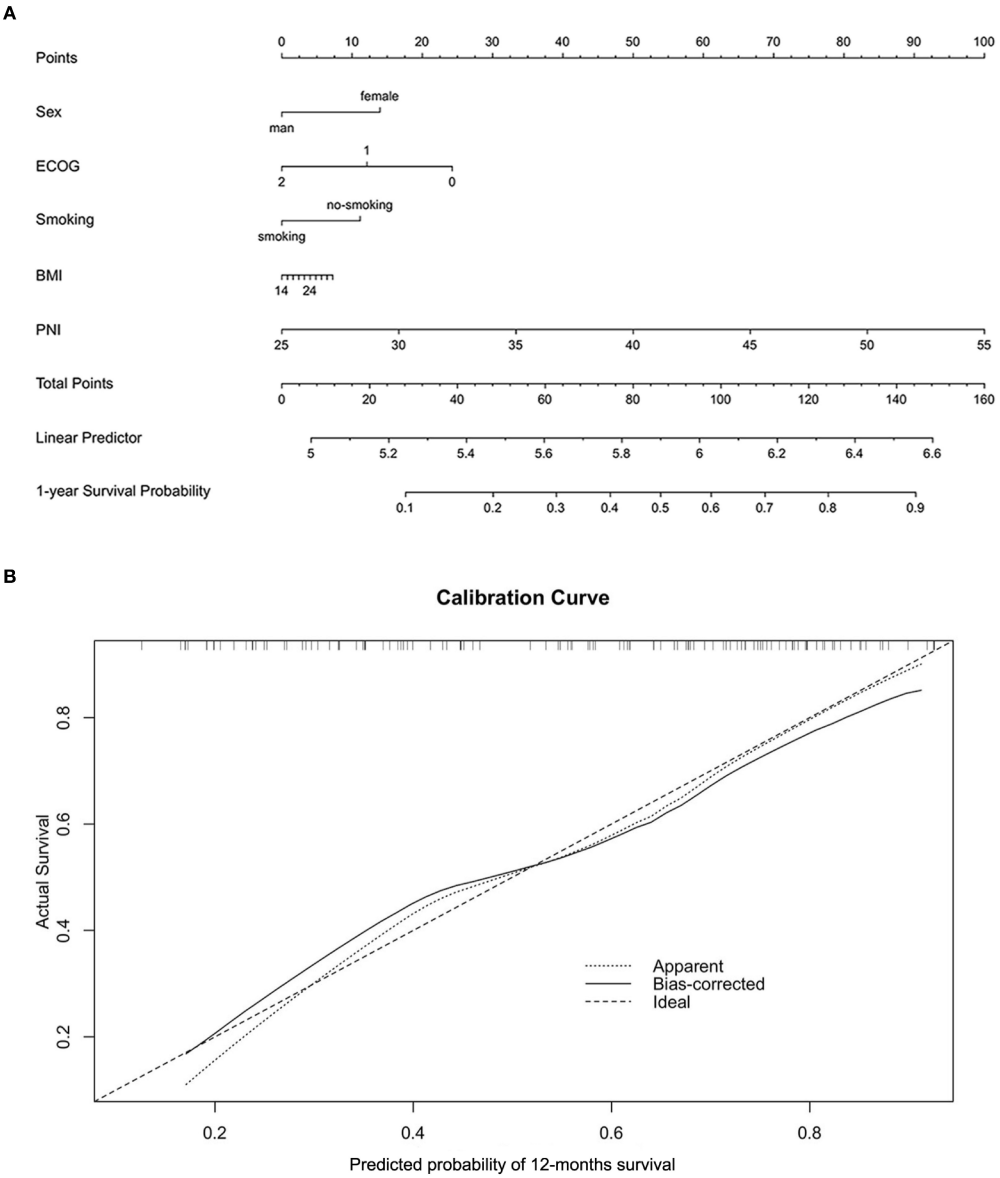
Construction and validation of a novel prognostic nomogram based on PNI. **(A)** A nomogram integrating PNI, gender, ECOG, smoking, and BMI was constructed to predict the 1-year survival probability of the entire validation cohort. **(B)** The calibration curve model was used to validate the accuracy of the nomogram in predicting survival probability.

For further investigating the prognostic impact of PNI, the subgroup analysis was carried out according to NSCLC (*n* = 72) or SCLC (*n* = 51). As shown in Supplementary Figures 1A, B, the survival analysis demonstrated the positive correlation of the PNI level with both the OS and PFS of the NSCLC patients. The result was then confirmed by the univariate analysis (Supplementary Figures 1C, D). Moreover, the multivariate analysis suggested the high PNI level was an independent favorable factor for both OS and PFS (Supplementary Figures 1E, F). The similar results were also observed in the SCLC subgroup (Supplementary Figures 2A–F). In addition, we noted that the patients with high PNI level had a significant better therapy response than those with low PNI level in both the entire cohort and subgroups stratified by cancer types (Figures 4A–C). The ROC analysis was also used to compare the predictive performance among PNI, BMI and albumin level (Figure 4D). The result demonstrated the PNI was superior to BMI or albumin level in predicting OS (AUC: 71.0% for PNI, 56.5% for BMI and 57.9% for albumin level). Finally, for better utilization of PNI in prognostic prediction, a nomogram was established based on PNI and other clinical parameters including gender, ECOG scores, smoking and BMI, which predicts the 1-year OS of ICI-treated lung cancer patients (Figure 5A). The calibration curve model was constructed to successfully validate the reasonable consistency between the predicted and actual survival (Figure 5B).

## Discussion

The ICI-based immunotherapy has significantly improved the overall prognosis of advanced lung cancer patients through its unique sustained tumor control and tolerable adverse effects (34). Since cachexia is common before or during anti-cancer treatment, therefore nutritional assessment and intervention are vital in patient management (35). The PNI, an easily available nutritional marker, has been utilized to predict the clinical outcome of the lung cancer patients in numerous studies. For instance, the low preoperative PNI level was proved to be an independent unfavorable factor affecting the OS and recurrence-free survival of the patients with resectable NSCLC (36). The high pretreatment PNI level was also correlated with better OS and PFS in advanced lung cancer patients receiving anlotinib (37). In extensive-stage SCLC, patients with high PNI levels had a significant better OS and PFS than those with low PNI level (38). However, on the contrary, a recent study found high PNI was associated with worse 1-year OS and therapy response in ALK-positive NSCLC patients receiving crizotinib, suggesting its prognostic impact may depend on gene mutations (39). With regard to ICI-treated lung cancer patients, whether the PNI could function as a reliable prognostic indicator remains inconclusive (16, 18, 25). Therefore, for addressing this issue, we performed a meta-analysis based on current evidences, followed by an independent clinical cohort validation. These efforts will contribute to the clinical utilization of PNI in the precise management of lung cancer patients.

In this meta-analysis, firstly, we found the high PNI level was significantly correlated with better OS and PFS of the lung cancer patients receiving ICI based therapies. The following sensitivity and publication bias analysis confirmed the stability and reliability of the results. This pooled result was in accordance with some included studies. For instance, in NSCLC patients receiving PD-1 inhibitors, PNI ≥ 45 was an independent favorable factor affecting OS and PFS (24). The similar result was also observed in another study suggesting low PNI level also served as an independent predictor of early progression (27). A recent report suggested the nutritional status may be correlated with the proportion and function of immune cells (such as T and memory cells) in the tumor microenvironment, therefore affecting the clinical efficacy of the immunotherapies (40). In hepatocellular carcinoma, nutrition deprivation impaired the anti-cancer function of tumor-infiltrating CD8+T cells, partly explaining why low PNI was correlated with reduced ICI efficacy (41). On the other hand, it should be noted that the PNI was not correlated with patient prognosis or identified as an independent prognostic indicator in several included studies (16, 18, 25, 28, 29). These divergent results may be partly attributed to some uncontrollable factors of retrospective studies such as sample size, patient selection, therapy strategy and follow-up period. For minimizing the potential impact of these factors, the subgroup analysis was performed based on specific characteristics. As result, the positive correlation between PNI level and OS/PFS was significant regardless of region, age and PNI cut-off value. However, with regard to therapy type, we found the PNI level was not significantly correlated with PFS instead of OS in the patients receiving anti-PD-1 therapy. This result was in accordance with only one of the included study demonstrating that pretreatment PNI, BMI and presence of sarcopenia were not identified as independent predictors for PFS in the NSCLC patients receiving pembrolizumab as the first-line treatment (31). Considering the limited included studies, we suggested the correlation between pretreatment PNI level and the clinical efficacy of anti-PD-1 drugs needs to be clarified by more evidences.

For validating the result of the meta-analysis, an independent retrospective cohort from two hospitals was utilized. For the whole cohort, the high PNI level was identified as a favorable independent prognostic factor for both OS and PFS, which was subsequently confirmed by the subgroup analysis of NSCLC and other lung cancer types. In addition, the high PNI level was found to correlate with better therapy response in the lung cancer patients. These evidences collectively supported the speculation that the pretreatment PNI level may predict the efficacy of ICI therapy in the lung cancer patients. On the other hand, both the meta- and retrospective analysis further highlighted the crucial role of immunonutrition intervention in cancer immunotherapy (42). Although the nutritional support in cancer neoadjuvant and adjuvant therapies was widely acknowledged and routinely performed, its benefits in ICI-based therapy remains unclear and needs to be clarified by more clinical trials (43, 44). Previous studies have suggested BMI has the potential to be a prognostic indicator in ICI-treated lung cancer patients. For instance, a multicenter study has showed BMI variation was significantly correlated with OS and PFS in metastatic NSCLC patients receiving first-line pembrolizumab treatment (45). A real-world clinical study demonstrated BMI⩾25 kg/m^2^ served as an independent favorable factor for the OS of ICI-treated NSCLC patients (46). Consistently, BMI > 25 kg/m^2^ was significantly correlated with longer PFS in EGFR mutated NSCLC patients receiving ICI therapy (47). The mechanism investigations attributed this correlation partly to the activated leptin signaling in overweight patients, which increases PD-1 expression to induce tumors more responsive to ICI drugs (48). In our study, we found the high BMI level was correlated with better OS and PFS in NSCLC patients, but failed to independently predict the clinical outcome in the multivariate analysis. A recent study has found the association between BMI level and the survival of ICI-treated patients may be affected by some clinical confounders, which partly explains the inconsistent results in our study (49). Moreover, a narrative review has implied the unreliability of BMI as a clinical indicator for lung cancer patients and recommended novel radiological strategies to define obesity (50). The serum albumin is recently identified as a novel non-invasive biomarker for predicting the clinical efficacy of ICI therapy in numerous cancers (21, 51). In this study, the increased serum albumin was correlated with better OS but failed to be an independent predictor in the ICI-treated NSCLC patients. Then, the ROC analysis demonstrated the PNI performed better in stratifying OS than the BMI and serum albumin, suggesting PNI may be the priority for clinicians in predicting the clinical outcome. Finally, considering the increasing utility of nomogram models in cancer management, we integrated the PNI and other factors to establish a novel nomogram for predicting 1-year OS. Using the calibration curves, the predictive performance of the nomogram was well-validated, further supporting the application of PNI in clinical practice.

There are some mechanism investigations to support the role of PNI in predicting ICI efficacy. The PNI value was calculated based on the serum albumin level and lymphocyte count, both of which are closely linked to the clinical efficacy of ICI drugs. The ICI therapy is dependent on IgG-antibody drugs and their catabolism and recycling are regulated by neonatal Fc receptor (FcRn) mediated mechanisms (52). The abundance and efficiency of FcRn could be reflected by the serum albumin level (51). The lymphocyte count may be correlated with the apoptosis of tumor infiltrating lymphocytes, which is crucial for determining the individual response to ICI therapy (53). On the other hand, considering the complexity of tumor microenvironment, the roles of albumin and lymphocyte in cancer immunotherapy are expected to be investigated by more fundamental work based advanced techniques such as single-cell sequencing. In addition, whether increasing pretreatment PNI level (such as albumin supplement or other nutritional interventions) is actually beneficial for the ICI therapy still needs to be clarified by well-designed prospective studies in future.

There are several limitations in our study. First, in the meta-analysis, the therapy strategies varied among the included studies, which may result in study heterogeneity. Meanwhile, the included literatures were relatively limited and therefore the results of the subgroup analysis need further validations. Secondly, a multicenter study demonstrated the PNI level was not an independent prognostic factor for the OS of the patients with SCLC, which is inconsistent with our retrospective study (29). Considering the few relevant published reports and limited sample size of our study, more attention should be paid to the prognostic value of PNI in ICI-treated SCLC patients. Thirdly, since the PNI and other parameters (such as BMI and albumin) may vary with disease progression, we failed to investigate the clinical values of their dynamic changes during the therapy period. Finally, our study mainly focused on the prognostic significance of pretreatment PNI in ICI-treated lung cancer patients, how to develop precise nutritional support strategies and their potential benefits on ICI efficacy should be emphasized in our following work.

## Conclusion

In conclusion, our findings collectively demonstrate the PNI level can be utilized as a reliable prognostic indicator for lung cancer patients treated with ICI therapy. These findings not only contribute to the clinical utility of PNI in cancer patient management, but also further highlight the crucial role of nutrition assessment and intervention in ICI therapy. Considering the study limitations, more clinical validations are needed in future. Moreover, relevant mechanism investigations may benefit developing novel intervention strategies to modulate PNI levels before or during ICI therapy.

## Data availability statement

The raw data supporting the conclusions of this article will be made available by the authors, without undue reservation.

## Author contributions

XY and JW wrote the main manuscript text. JM and YW collected the data and prepared all the figures. HQ analyzed the data and revised the manuscript text. XW and MY designed the study and revised the manuscript text. All authors reviewed the manuscript. All authors contributed to the article and approved the submitted version.

## Supplementary material

Supplementary Figure 1 Prognostic significance of prognostic nutritional index (PNI) in the patients with non-small cell lung cancer (NSCLC). **(A, B)** Kaplan-Meier curves for the association of PNI with overall survival (OS) **(A)** and progression-free survival (PFS) **(B)** of the NSCLC subgroup. **(C, D)** Univariate analysis for identifying the factors significantly correlated with the OS **(C)** and PFS **(D)** of the NSCLC subgroup. **(E, F)** Multivariate analysis for identifying the significantly independent factors affecting OS **(E)** and PFS **(F)** of the NSCLC subgroup.

Supplementary Figure 2 Prognostic significance of prognostic nutritional index (PNI) in the patients with small cell lung cancer (SCLC). **(A, B)** Kaplan-Meier curves for the association of PNI with overall survival (OS) **(A)** and progression-free survival (PFS) **(B)** of the SCLC subgroup. **(C, D)** Univariate analysis for identifying the factors significantly correlated with the OS **(C)** and PFS **(D)** of the SCLC subgroup. **(E, F)** Multivariate analysis for identifying the significantly independent factors affecting OS **(E)** and PFS **(F)** of the SCLC subgroup.
