# Supplementary figures and images for: Identification of prognostic nutritional index as a reliable prognostic indicator for advanced lung cancer patients receiving immune checkpoint inhibitors
Source: Front Nutr. 2023 Jul 28;10:1213255. doi: 10.3389/fnut.2023.1213255 (PMC10416798; doi:10.3389/fnut.2023.1213255)

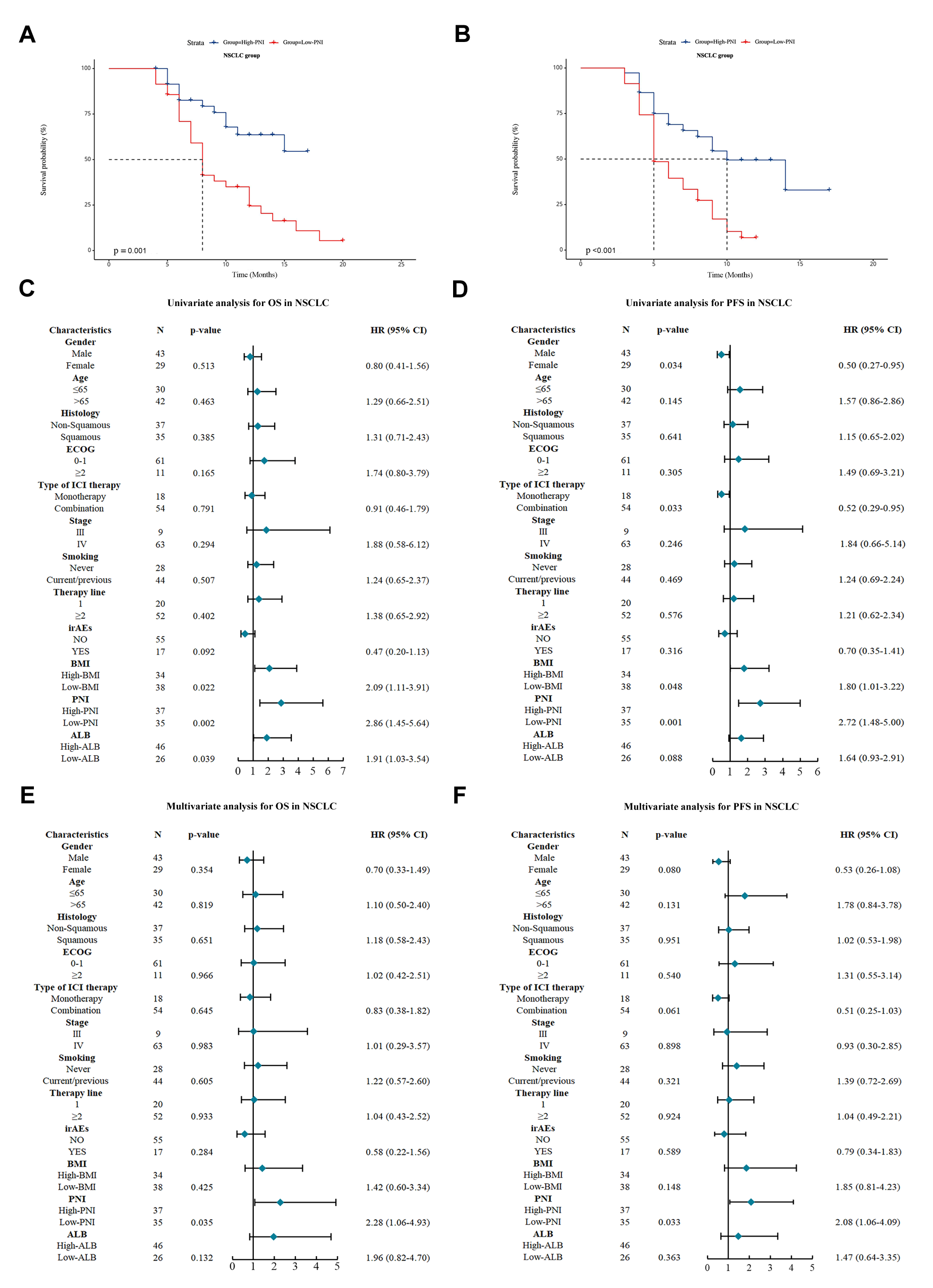

Supplement: Supplementary Figure 1 — Prognostic significance of prognostic nutritional index (PNI) in the patients with non-small cell lung cancer (NSCLC). (A, B) Kaplan-Meier curves for the association of PNI with overall survival (OS) (A) and progression-free survival (PFS) (B) of the NSCLC subgroup. (C, D) Univariate analysis for identifying the factors significantly correlated with the OS (C) and PFS (D) of the NSCLC subgroup. (E, F) Multivariate analysis for identifying the significantly independent factors affecting OS (E) and PFS (F) of the NSCLC subgroup. [file Image_1.TIF]

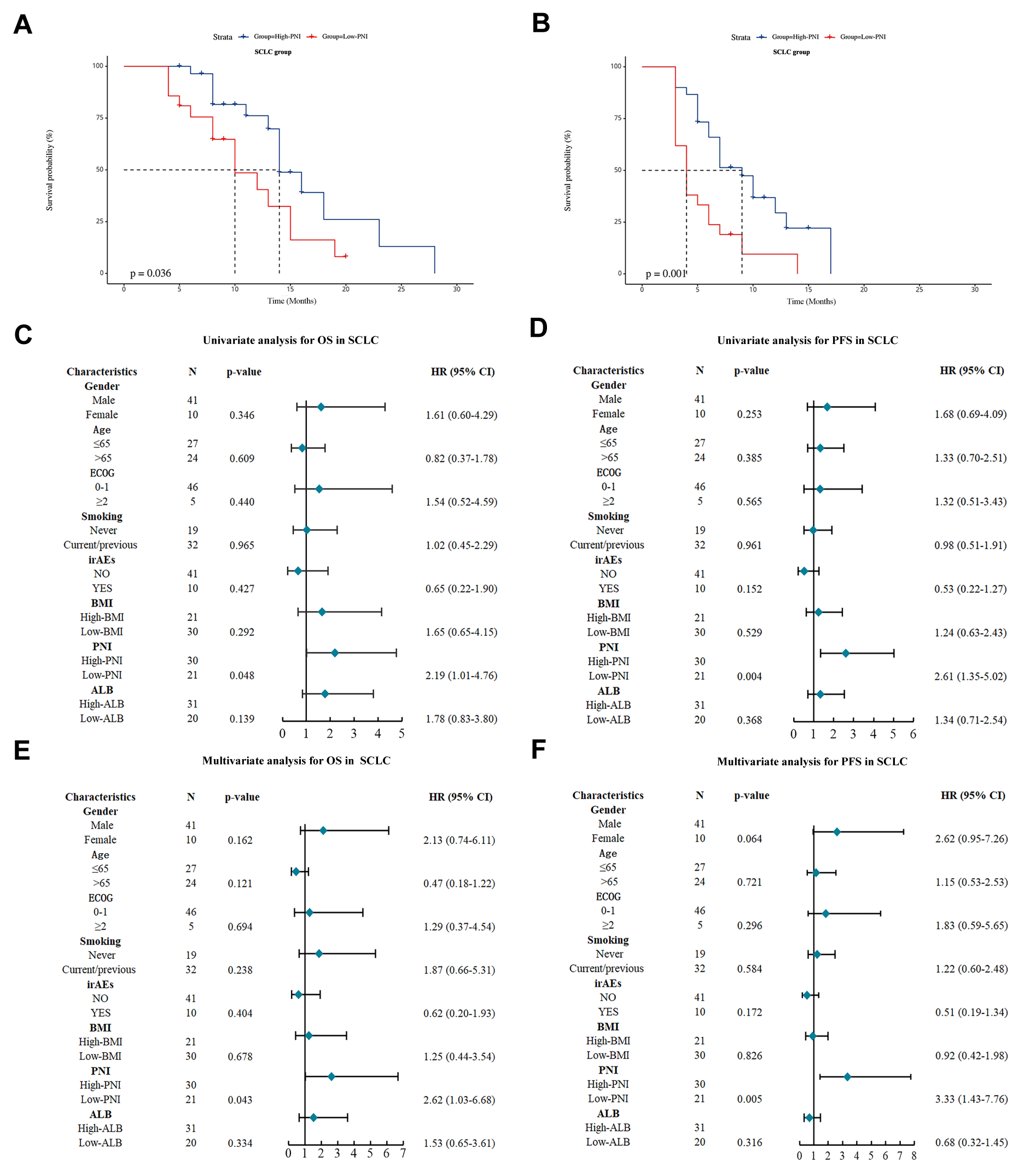

Supplement: Supplementary Figure 2 — Prognostic significance of prognostic nutritional index (PNI) in the patients with small cell lung cancer (SCLC). (A, B) Kaplan-Meier curves for the association of PNI with overall survival (OS) (A) and progression-free survival (PFS) (B) of the SCLC subgroup. (C, D) Univariate analysis for identifying the factors significantly correlated with the OS (C) and PFS (D) of the SCLC subgroup. (E, F) Multivariate analysis for identifying the significantly independent factors affecting OS (E) and PFS (F) of the SCLC subgroup. [file Image_2.TIF]
